# Computerized database management system for breast cancer patients

**DOI:** 10.1186/2193-1801-3-268

**Published:** 2014-05-27

**Authors:** Kok Swee Sim, Sze Siang Chong, Chih Ping Tso, Mohsen Esmaeili Nia, Aun Kee Chong, Siti Fathimah Abbas

**Affiliations:** Faculty of Engineering and Technology, Multimedia University, Jalan Ayer, Keroh Lama, 75450 Melaka Malaysia; Melaka General Hospital, Jalan Peringgit, 75990 Malacca Malaysia

**Keywords:** Breast cancer patients, Computerized database, Hospital management system

## Abstract

Data analysis based on breast cancer risk factors such as age, race, breastfeeding, hormone replacement therapy, family history, and obesity was conducted on breast cancer patients using a new enhanced computerized database management system. My Structural Query Language (MySQL) is selected as the application for database management system to store the patient data collected from hospitals in Malaysia. An automatic calculation tool is embedded in this system to assist the data analysis. The results are plotted automatically and a user-friendly graphical user interface is developed that can control the MySQL database. Case studies show breast cancer incidence rate is highest among Malay women, followed by Chinese and Indian. The peak age for breast cancer incidence is from 50 to 59 years old. Results suggest that the chance of developing breast cancer is increased in older women, and reduced with breastfeeding practice. The weight status might affect the breast cancer risk differently. Additional studies are needed to confirm these findings.

## Introduction

Computerized database management (CDM) system has been widely implemented in many hospitals over the world to allow the proper management of medical records for different types of cancer patients (Ann et al. 2003). In Malaysia only a small number of hospitals have implemented a CDM system, due to the high installation and implementation cost and the lack of trained technician for maintenance. Medical information is needed in any health organization to avoid medical errors and inappropriate decisions. CDM based management systems have been developed to address the increasing demand of accessibility of medical information.

In CDM systems, patients’ electronic medical data are collected and recorded to offer enough information and support to physicians for medical decision making. Different CDM models have been developed and used during the past decades (Delpierre et al. 2004). Walton et al. (1997) evaluated the computer support for general practice and prescribing (CAPSULE) using simulated cases. Castelden et al. (1988) developed a computer-based discharge and data-collection system for surgical audit. Safran et al. (1995) examined a CDM system on HIV infection, where their developed system works as the guidelines for management of HIV infection with computer-based patient’s record. The common characteristic of all these systems is in assisting and providing patients electronics medical records to physicians and improving quality of care.

Adusei et al. (2010) developed an intelligent CDM system for a computer aided breast cancer detection and diagnosis using mammography, where the CDM system efficiently retrieves and analyzes the mammogram images. The aim of CDM systems is to provide a fast and secure platform to health care organizations, to improve the speed of simultaneous access and retrieval of medical records, and improving confidentiality of medical records (Delpierre et al. 2004).

The motivation of the present work is to devise an appropriate CDM system, specifically for breast cancer patients in Malaysian hospitals, although the product can be adapted to other patients or countries. The first part of the paper describes the implementing of an analytic database management system. The system provides the functions such as storing and retrieving the patient data, inserting new patient data, updating or deleting the data, and appointments schedule. The second part of the paper describes the breast cancer patient data analysis. An automated calculation tool is developed in the analytic database management system for this purpose. Analysis on several breast cancer risk factors such as age, race, breastfeeding, hormone replacement therapy, family history and obesity are performed.

Microsoft Access and MySQL are the tools that can be used to implement the relational database management system. Relational database is a collection of data items where the data are organized into the table form, and data can be accessed in many different ways without reorganizing the database tables (Allen 2006). This database management system has the capability to gather, store and transmit the medical record information from different sites of hospital to a centralized database system (Kouji et al. 2000).

Microsoft Access is a popular data management application that enables the storage of information or data in tables that it manages from the local disk (Paul 2011). Microsoft Access is able to develop a ‘back-end’ database to hold the desired data while maintaining a user-friendly ‘front –end’ interface (Kouji et al. 2000). The easy to use front-end interface is one of the advantages of Microsoft Access. Microsoft Access also provides a replication feature that allows data transmission, and combines it into a single master database. Access allows data in multiple copies of a single database to be maintained in high synchronicity along all the copies. During data entry, the records, which are entered, updated and deleted are observed and tracked by the replication feature. There are several limitations of Microsoft Access. It is generally used as a personal or single user application, typically for managing and organizing of limited amount of data (Paul 2011). There is storage size limitation for Microsoft Access database. As a result, Microsoft Access is not commonly being used for large databases.

### Issues in existing practice in Malaysia

There are a lot of patients seeking diagnosis and medical treatment of breast cancer in hospitals every day. As an example, at the Melaka General Hospital (MGH), the current practice is to handle the huge amount of data through the hardcopy format. First, the patient registration is done in hardcopy format. Physician conducts the breast examination, and a suitable breast screening test such as mammogram, the physician may suggest ultrasound, or breast biopsy if the noticeable symptoms are found. A hardcopy report which composes the description of patient’s condition and type of screening test is sent to the radiology department in the hospital. After the test, the images and results, which are in hardcopy format as well, will be transmitted to the physician, who will then decide on future action. All the hardcopies are stored in the documentation room.

The current data management system in MGH has a low security level. All medical reports stored in documentation room are easily accessible by unauthorized people. Besides, during the delivery of reports between various departments, duplication, thefts, and misplacements are not uncommon. Moreover, there is no information control for separation between the general staff and the physicians. Apart from security issues, physical transfer of reports is time consuming. This can be important issue when a medical report is needed urgently. In addition, due to the large amount of information stored in the documentation room, and complex arrangement of records that can hamper retrievals, updating or altering the records is difficult and time consuming.

From cost point of view, the hardcopy format is considered a variable cost that increases as the patient number increases. Manpower is required to transfer and to organize the reports. The storage problem is also a cost issue and increases with the age of the hospital. The alternative way in discarding old reports causes a loss in valuable information that could be used for data analysis or for historical comparative studies.

Furthermore, when medical reports are lost or damaged, it is almost impossible to retrieve the lost information. Unlike a computerized system which can perform routine partial checks, human errors incurred in filling the medical forms and reports are more difficult to be detected by the system, since the report must be seen by another person before the abnormally can be discovered.

Analysis of data enables useful information to be discovered which may help in medical studies and medical decision making. However, this is a daunting task to be accomplished if reports are in hard copies, where all the analysis needs to be performed manually. The accuracy of the analysis is also not as good as computerized calculations.

### An analytic database management system for breast cancer patients

#### The objectives

To overcome the limitations of the existing system, an analytic database management system is proposed that allows data to be collected, stored, updated and retrieved easily. Table 1 gives the descriptions on the proposed solutions to overcome the described problems.

**Table 1:** **Descriptions of proposed system**

Criteria	Descriptions	Proposed features
High security	Require username and password in order to gain access into the system.	User login page
	Provide differential accessible level for the user, where not all functions can be performed by some users.	Main menu page
	Indicate dates of modifications and updates.	Patient personal details and diagnosis report page
Time saving	Link several computers in the hospital together in order to allow the transfer of reports or data.	Server and client network
	Provide a feature that can rapidly view, retrieve, update and modify the database.	Patient personal details and diagnosis report page
Low cost and less man power	Low cost as all data are stored in the database rather than on hard copies.	MySQL database
	Require less man power as the database can be easily handled.	MySQL database
Unlimited storage	The MySQL database has near unlimited storage capability.	MySQL database
Less human error	All the data are stored in the permanent database with back-ups. Data will not be easily lost or erased.	MySQL database
	Feature allows user to insert the new patient data during patient registration and there is a notification given for missing information.	New patient registration page
Fast and easy to perform data analysis	Automatic calculation tools to assist data analysis and output graphs are plotted automatically.	Data analysis page

### Details of design

The system is designed specifically for handling and managing medical information such as personal details and diagnosis report used in breast cancer department and required data analysis with ease and comfort. Medical records are collected and stored in large databases. A graphical user interface (GUI) is created to work as a user friendly ‘front-end’ interface while controlling the ‘back-end’ database, and serves as the communication bridge between user and database.

The features that have been included in the databases management system architecture are shown in Table 2.

**Table 2:** **Features of analytic database management system**

Features	Description
User login	• Provide different level of accessible users such as staff and doctors, with password requirement.
New patient registration	• Allow the registration of new patient to be done digitally without filling any hardcopy form. The information is stored directly into the database.
Patient personal details	• Enable the viewing of all details in the department.
	• Provide update, edit, and delete functions which allow modifications to be done.
Patient diagnosis report	• Hold the diagnosis reports for all patients.
	• Allow the authorized user to view, edit or update the diagnosis reports for certain patients
Appointment	• Allows the search and view appointments.
	• Assist staff to arrange appointments for patients and doctors.
Data analysis	• Distribution of patients based on their age and race.
	• Perform analysis on patients with or without breast cancer.

### Data and database

The design of database is highly dependent on the type of stored data and the method of data collection. In this project, data such as patient details and diagnosis reports are to be stored, as shown in Table 3.

**Table 3:** **Overall patient data**

Data	Content
Personal details of patient.	Registration number, identification card number, name, age, marital status, section, race.
Patient’s background.	Breastfeeding, family history, hormone replacement therapy.
Noticeable symptoms of breast cancer found.	Pain, mass, discharge.
Type of screening tests that had been performed.	Mammogram, ultrasound, MRI, breast biopsy.
Type of image guidance device for breast biopsy.	Ultrasound, stereotactic mammography, hook wire.
Type of operations to be performed if needed.	Biopsy, mastectomy, excision.
Diagnosis reports.	Mammogram report, ultrasound report, MRI report, breast biopsy report.

**Figure 1:**
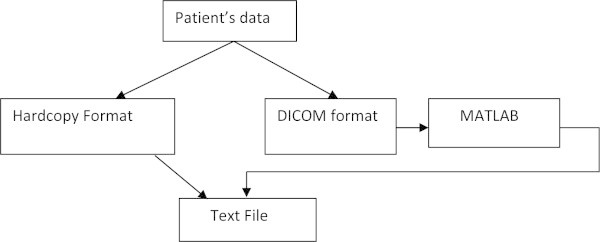
**Block diagram for creating a text file.**

For this project a total of 1057 patient data (mainly in hardcopy format) for breast cancer are collected from MGH. As a special case, a patient who is examined under MRI have the data normally saved in digital image and communications in medicine (DICOM) format, a standard for managing, storing and transferring the information in medical imaging. DICOM files can be exchanged between two entities such as retrieving the images or obtaining patients’ data (Kimura et al. 1998).Since the formats of the data are different, a standardized format of data is necessary. For data, which are stored in hardcopy format, a text file is created in order to convert the patient’s data into softcopy format. On the other hand, information stored in the DICOM file need to be retrieved and stored into the text file together with the corresponding MRI diagnosis report which has been converted to the softcopy format. In order to read the DICOM files, MATLAB programming language is used, as illustrated in Figure 1.

**Figure 2:**
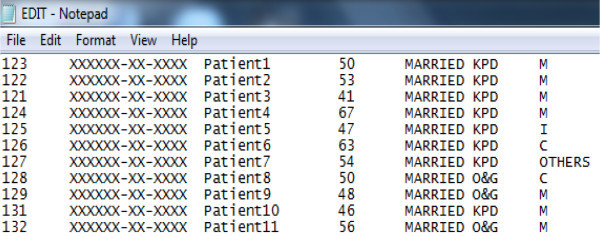
**The text file created.**

**Figure 3:**
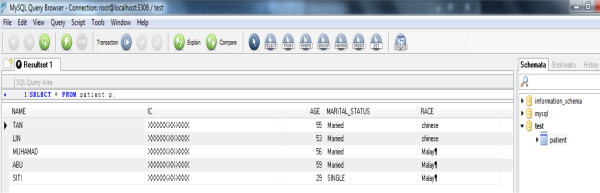
**MySQL database.**

Figure 2 shows a sample text file. The text file holds all the information listed in the Table 3, and stored in DICOM files. They are loaded and stored in the database.The database is a systematic and organized storage medium for handling and managing the information. MySQL was chosen as the essential system program to implement the system database for its low cost and adequate security. MySQL provides a fast, flexible, secure and stable medium for retrieving, updating and entering the information into the database by an authorized user. The developer can design and manipulate the data in their desired way using a structured query language (SQL) with statements that are part language, part mathematics. Figure 3 shows the result of execution of the LOAD DATA command. All the data in the text file are successfully transferred.

Although MySQL programming is not a complicated programming language, time and effort need to be contributed in order to build up the good and strong foundation in the programming language. As a result, the limitation of MySQL database is the low usability.

### Concept of GUI

**Figure 4:**
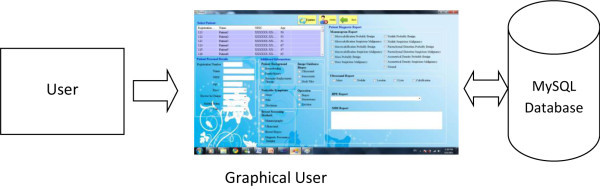
**Concept of GUI.**

**Figure 5:**
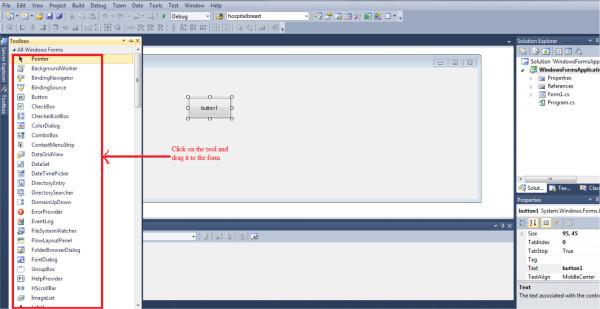
**Dragging the tool into the form.**

**Figure 6:**
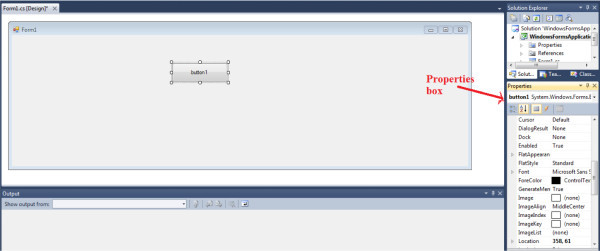
**Properties box.**

The graphical user interface (GUI) is the communication bridge between user and the database, and provides ease of use and interaction abilities for the user of MySQL database. For instance, the user can easily update records, insert new records or delete the existing records in the MySQL database by just clicking on the particular features in the GUI such as a button or a checkbox. There is no need to learn any programming skill. Figure 4 illustrates the concept of GUI interface.There is a list of tools which are available at the left. The desired tools such as button, text box, group box and list view can be dragged from the toolbox into the form side in order to create the GUI. The list of tools which can be found in the toolbox is shown in Figure 5.A properties box is placed at the lower right of the Window Application Form 1. It allows the modification on the tool properties such as the size, text, font, color, and the visibility. Figure 6 shows the properties box which appears after a button is dragged into the form side.

All the features listed in Table 4 are included in the GUI interface design. In order to enhance the communication with the user and importing the information into the database, necessary features with several input data tools as well as button, input textbox, and checkbox, are implemented on the single form side.

**Table 4:** **Common tools implemented in the design of the GUI Interface**

Tools	Description	Example
Button	Perform an action as describe in the codes.	
Textbox	Enables user to insert text, provides multi-line editing and password character masking.	
Checkbox	Allows the user to select or clear the particular option.	
List view	Display a collection of associated items and allow the user to select on the particular item.	
Group box	Display a frame around the group of associated tools.	

### Main menu page

The main page access features a list of links for visiting different pages such as patient registration page, personal details, diagnosis report, statistical analysis and appointments. In order to enhance the security of the system, the accessibility level into the pages is based on the user type, for instance, a nurse or staff can have access to the information but only the physician can update or edit the patient report.

#### Design of assessibility

User can access the desired page by clicking on the relevant button. The main menu page is formed by grouping and framing the buttons within a group box. Initially, the group box’s visibility for the main menu feature is set to “false” in the properties box. Once the access is gained, the visibility is changed to “true”.

#### Patient registration

This page is accessible by the nurse or staff only for inserting new patient records into the database. In order to reduce human error and avoid the data lose, a notification is given when important data are not inserted. There are several checkboxes, which enable the user to select the patient’s background and symptoms of breast cancer. A button is used to send all the input data into the MySQL database if it is activated. All input controls are framed with a group box in order to form the new patient registration page.

#### Codes

**Figure 7:**
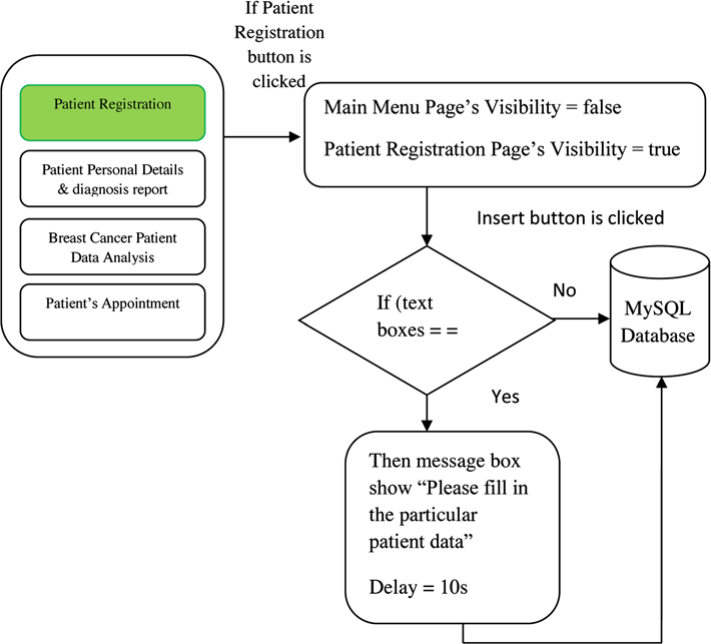
**Patient registration code flowchart.**

Initially, the group box’s visibility is set to false in the properties box. If the button for accessing to this page is clicked by the user, the group box visibility is set to true in the codes whereas the group box‘s visibility for main menu page is changed to false in the codes. The patient information is filled in the particular input text boxes and checkboxes. If the insert button is clicked, there is a sequence of actions being performed. First, the textboxes for inserting the certain important patient information are checked. If the text boxes are empty then a message box appears as a reminder to fill in the missing information. A delay system is implemented while waiting for the delayed response. Then, connection to MySQL database is established. The MySQL insert statement is written for sending the new patient information from the textboxes and checkboxes into the table of MySQL data. Figure 7 shows the patient registration code flowchart.

### Personal details and diagnosis report

**Figure 8:**
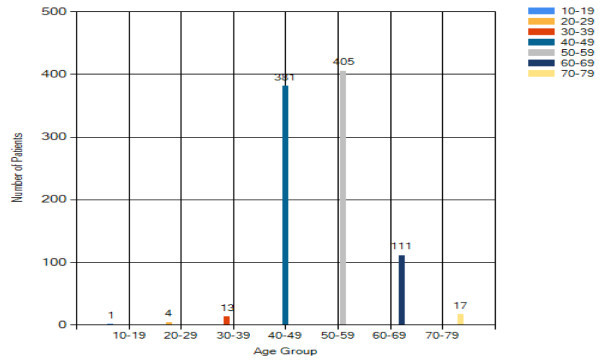
**Benign breast change analysis result – age.**

A further enhancement for the security of analytic database management system is accomplished through recording date and time that the patient data are updated or edited in MySQL database and then displayed on the GUI. Figure 8 shows the patient personal details and diagnosis code flow.

#### Design

**Figure 9:**
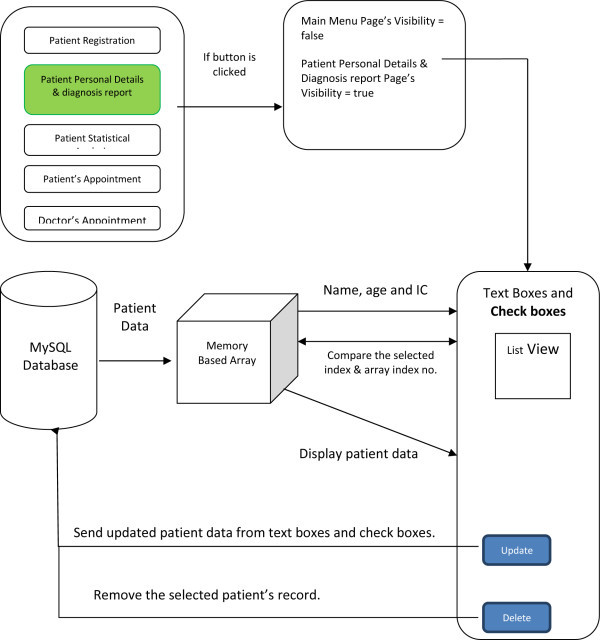
**Patient personal details and diagnosis code flow.**

To view, update and edit functions of the patient personal details and diagnosis report, the user can select the patient’s name from the list view. The selected patient’s data will be shown on the text boxes and check boxes. To edit the patient personal details and diagnosis report, the contents of text boxes can be modified or edited and checkboxes can also be selected or cleared. After the modifications the update button is used to store the up-dated information into the MySQL database.To delete a patient’s record, selecting and click on the delete button. All the features are gathered by the group box to form the page. Figure 9 shows patient personal details and diagnosis code flow.

### Breast cancer patient data

The feature allows the distribution on patients based on their age and race, categorizing into groups based on their diagnosis reports. Further information regarding breastfeeding, family history and hormone replacement therapy are also included. The weight status of the patients are distributed into normal weight, overweight and obesity groups, according to the body mass index calculated by using the Eq. (1), and guided by Table 5.

**Table 5:** **Body mass index standard**

Normal weight	18.5 ≤ Body mass index <25
Over weight	25 ≤ Body mass index <30
Obesity	Body mass index ≥ 30


                    
                        1
                        
                    
                

### Client network

**Figure 10:**
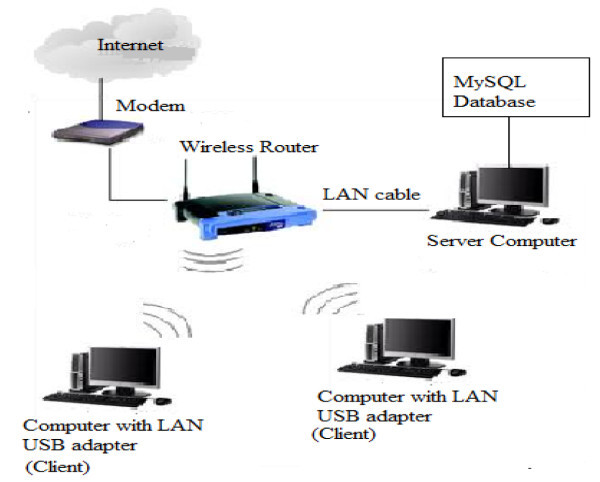
**Illustration of the connections among the components.**

The analytic database management system is installed into several computers in the Hospital in order to support multiple accesses and multiple task performance. For instance, the analytic database management system is implemented at the patient registration counter in order to insert new patient information into the database. After the registration is completed, the doctor is able to obtain the new patient information from the system which is installed in his/her computer. A centralized MySQL database system enables several computers direct access to database for storing, retrieving and updating via network. This is done by setting up a computer as the server to host the MySQL database as shown in Figure 10.

With wireless local area network (LAN) technology, the MySQL database server computer and each of the clients can be easily linked together. The access into the MySQL database in the server is allowed by declaring the unique IP address of server in the system programming part installed in each client.

### Breast cancer data analysis

The patient page that shows patient personal details and diagnosis report is linked with the new patient registration page that doctors can have access to. However, diagnosis reports that are edited by the nurse or staff will not be updated in the MySQL database. The date and time for the update action will be captured automatically and recorded in MySQL database. There are four analysis topics that can be selected: overall analysis, breast cancer patient analysis, benign breast changes patient analysis and screening method analysis.

### Overall analysis

**Figure 11:**
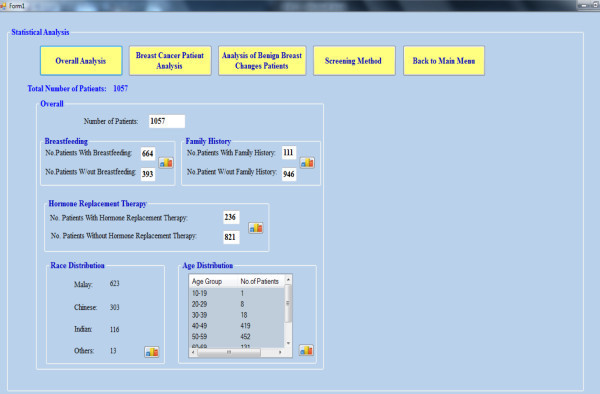
**Overall analysis.**

On clicking the overall analysis button, the overall analysis is displayed as shown in Figure 11.

The overall analysis is the study of the several factors such as age, race, breastfeeding, family history and hormone replacement therapy for all patients, regardless of whether there is a development of breast cancer or not. All these factors are considered as the changeable and unchangeable factors that tend to affect the breast cancer risk for patient. Of the 1057 patient data stored in the Hospital, not all patients had the development of breast cancer. By clicking on the small icon inside each group box, the graph for each factor is plotted on the right hand side of the page. The result and discussion for each factor will be explained.

#### Breastfeeding

**Figure 12:**
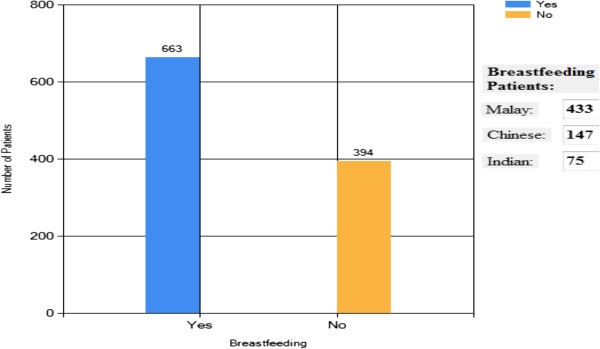
**Overall analysis result – breastfeeding.**

Figure 12 shows that among the 1057 patients, 63% have breastfeeding, 395 patients do not breast their infants. Among 663 patients, 533 are Malay. This result may reflect that most of the Malays breastfeed their infants as compare to other races (Vanzo et al. 1994).

#### Family history

There is small number of 111 patients with family history of breast cancer among 1057 patients. This is decided from the interview conducted during registration, the patients who meet the requirements as described earlier are considered to have family history of breast cancer. As family history factor is an unchangeable factor and is highly depending on the inherited DNA, there is no relationship between race and family history. So far, there is no known connection between the two.

#### Hormone replacement therapy

From the results, there are 236 patients who undergone hormone replacement therapy, representing 22% of overall patients. This result may imply that the therapy is not well accepted by Malaysians. The reason may be due to the fear that hormone replacement therapy tends to increase breast cancer risk.

#### Race

**Figure 13:**
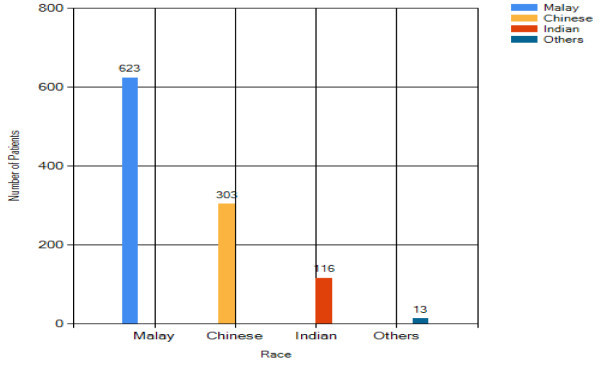
**Overall analysis result- race.**

As seen in Figure 13, almost 60% of patients in the breast cancer department are Malay whereas 40% are Chinese and Indian. This result is considered normal as the majority population in Malaysia is Malay.

#### Age

**Figure 14:**
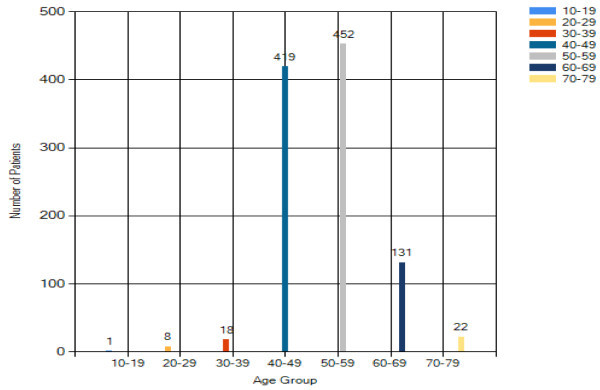
**Overall analysis result – age.**

Of the 871 patients who seek medical consultation on breast cancer, results in Figure 14 reflect that 83% of the patients fall in the 50–59 age groups. This may be connected to the reduction in female hormone production during the menopause period in this age group.

### Breast cancer analysis

As mentioned earlier, there is a GUI feature designed to distinguish between breast cancer or benign breast change based on the breast biopsy results. Among 1057 patients, there are 71 diagnosed with breast cancer based on the analysis of breast biopsy result. The analysis is based on several breast cancer risk factors such as breastfeeding, age and race may allows the discovery of useful finding which aids in the prevention and awareness of breast cancer. The results and discussions for the analysis each risk factor in breast cancer patient are next discussed.

#### Breastfeeding

**Figure 15:**
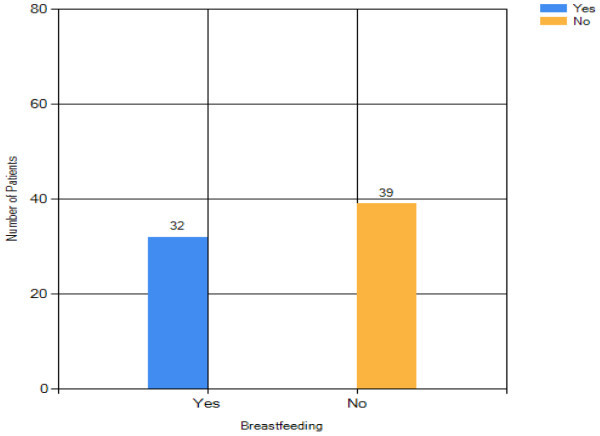
**Breast cancer patient analysis result - breastfeeding.**

Figure 15 shows that among the breast cancer patients, 32 breastfed their infants. There is very small deviation in the number of patients with and without breastfeeding reflecting that there is an inverse association between breastfeeding and breast cancer risk. This finding is in an agreement with several studies, which perform the investigation on the effect of breastfeeding on breast cancer risk (Hisham and Cheng 2003; Yip and Ng 1996; Su et al. 2010). More information such as duration of breastfeeding, sufficiency of milk and the age of first time breastfed can be collected in the interview section during the patient registration in order to support the analysis on the relationship between breastfeeding and breast cancer risk.

#### Family history

There are six patients with family history of breast cancer among the total 71 breast cancer patients. These patients are categorized into the high risk of breast cancer group.

#### Breast cancer patients analysis – hormone replacement therapy

There is small number of 5 breast cancer patients who accepted hormone replacement therapy. Although some studies had stated that hormone replacement therapy can increase the risk of breast cancer, this statement could not be verified as the number of patients is too small.

#### Breast cancer patients analysis – race risk factor

61% of breast cancer patients are Malay whereas Chinese contribute 27% of total breast cancer patients and the remaining 12% are Indian. This result shows that in this hospital, the incidence of breast cancer among Malay ethnic group is higher than other ethnic groups, and concurs with another study (Hisham and Cheng 2003). The result shows that there is low incidence rate of breast cancer among Indian women. This may be due to the diet, as there are some studies which stated that the spices in Indian cooking, especially “turmeric”, has the protection against breast cancer. The frequent intake of food which high in nuts and fibers and reduction in the consumption of sweet food can help in decreasing the breast cancer risk (Yip and Ng 1996; Su et al. 2010). Based on the analysis between race and age of breast cancer patients, majority of Malay and Chinese breast cancer patients are from 50 to 59 years old.

#### Breast cancer patients analysis – age risk factor

**Figure 16:**
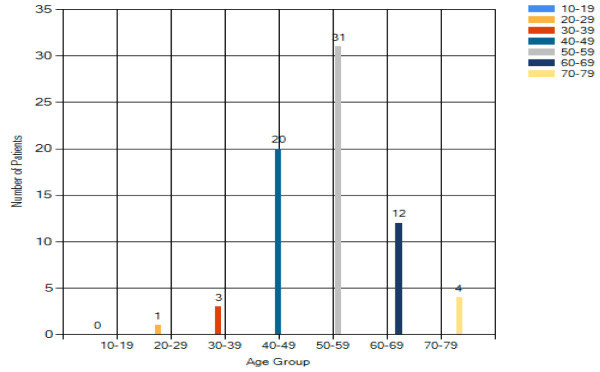
**Breast cancer patient analysis result – age.**

From Figure 16, the peak age group for breast cancer is 50–59 years old. This reflects that Asian tend to have earlier incidence of breast cancer when compared with the Caucasian (60 to 65 years old (Yip and Ng 1996)). Results also show that older women are more vulnerable to breast cancer. It is also believed that menopause could be a factor statement.

#### Breast cancer patients analysis – obesity risk factor

Obesity may be one of the risk factors for breast cancer. Among 71 breast cancer patients, there are only 15 breast cancer patients’ height and weight given by hospital authority. Among these 15 patients, 12 are obese. The majority of the 12 obese patients are from 50 to 59 years old. The result shows that increase in body mass tends to cause an increase in risk of breast cancer among older patient (>35 years old), in agreement with a study (Erin et al. 2010). The finding may help to increase awareness among women regarding importance of maintaining a healthy weight. The overweight also has the greater risk of breast cancer recurrence or death [Su et al. 2010; American Cancer Society 2011; Peacock et al. 1999; Zografos et al. 2004; Zheng et al. 2001).

### Analysis of benign breast changes

**Figure 17:**
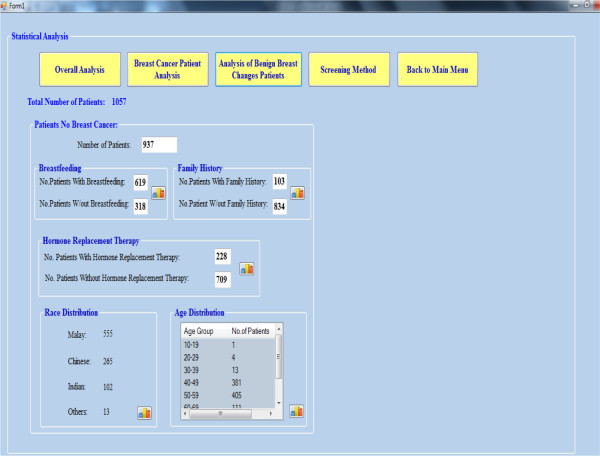
**Analysis of benign breast change patients.**

Although benign breast changes are non-cancerous, some types such as atypical lobular and ductal hyperplasia do increase the cancer risk. Among the 1057 patients, there are 937 patients with benign breast changes. Figure 17 shows the analysis page.

#### Benign breast change analysis – breastfeeding

Among the patients with benign breast changes, a majority of 66% patients had breastfeeding. There is large deviation between the patients with and without breastfeeding.

#### Benign breast change analysis – family history

There is small number of benign breast changes patients with family history (11%). This result may show that the occurrence of benign breast changes is not related to the family history of breast cancer.

#### Benign breast change analysis – hormone replacement therapy

25% of the total benign breast change patients had been used hormone replacement therapy. The number of benign breast changes patients had been used hormone replacement therapy is slightly higher than the breast cancer patients (7%). Figure 17 shows benign Breast Changes Analysis Result – Hormone Replacement Therapy.

#### Benign breast change analysis – race

**Figure 18:**
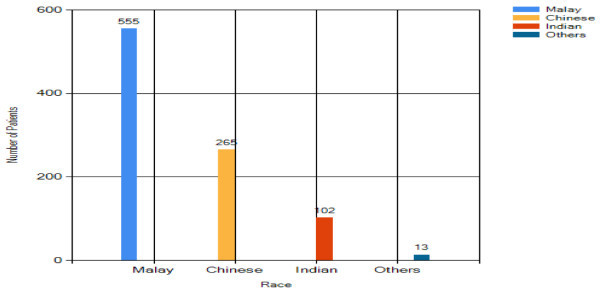
**Benign breast change analysis result – race.**

For the race distribution analysis in Figure 18, the majority of benign breast changes patients are Malay (59%). The number of Chinese with benign breast changes is half of that of Malay. The Indian is the lowest ethnic group (11%).

#### Benign breast changes analysis – age

Majority of benign breast changes patients are with age from 50 to 59. The result in Figure 8 shows that the reduction of female hormone production before or after menopause tends to cause some changes on the breast in both physical appearance and internal activities.

### Screening methods analysis

**Figure 19:**
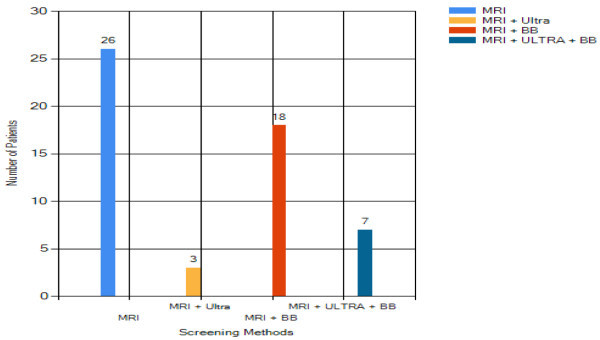
**Patients undergoing selective screening methods.**

Four breast examination methods, mammography screening, ultrasound screening, breast biopsy and MRI screening are developed for the breast cancer detection purpose. Among 1057 patients, 1003 undergo mammography screening test. As mammography is not ideal, another screening test is required if cancer is in suspect. This could be an ultrasound test, an MRI test or a breast biopsy test. Figure 19 shows the results.

There are 55 patients categorized into high risk of breast cancer. For them, the mammography screening test is skipped and they will be examined under breast MRI. If the MRI screening test is still undetermined, ultrasound or breast biopsy is taken.

This analysis provides useful information which might help in reducing screening cost. For instance, the breast structure (dense breast reduce mammography sensitivity) and patient age (age 35 to 50 can be screened under MRI) should be analyzed before selecting the screening method. This is because if the correct screening method is chosen, the diagnosis can be obtained accurately, saving the cost of extra screenings.

From the physician’s experience, a satisfactory user experience was reported regarding the use of CDM, with some concern regarding its limitations.

The impact on the physician-patient relationship and physical limitations and barriers are between two main reported concerns on the use of CDM. These limitations could influence the use of the CDM system during the consultation.

Kaushal et al. (2003) showed that CDM systems could avoid prescription errors. To develop an accurate CDM system, and improve physician-patient consultation quality, the quality of collected data is very important and essential, and should be controlled and certified. Considering the risks, possible instability and data loss in computer based databases; it is a common practice to keep multiple secure backups of patient records. While most of patients are satisfied with the use of CDM, and their main concern is the confidentiality of their records, more studies might be required to analyze the quality of use CDM during consolation, and patients feeling and concerns regarding the CDM system. The impact of CDM use on preventive care was clearer with and more balanced.

## Conclusions

An analytic database management system has been proposed and developed in this project. This system not only provides the facilities for storing, retrieving and updating breast cancer patient data but it also provides the tools which assists the data analysis to be performed on the stored data. In order to develop the analytic database management system, 1057 breast cases data in General Hospital Melaka have been collected and stored in the new system. There is a GUI interface, created to provide user friendly interface while controlling the database management system. The GUI interface acts as the connector between the user and database. Functions such as update, edit, delete and data analysis had been defined on the GUI interface.

The analysis has obtained some useful findings. Among 1057 patients, majority of them are Malay, followed by Chinese and Indian, there are 71 patients diagnosed with breast cancer. Most of the breast cancer patients are between 50 to 59 years old. This result shows that as the women are older, the chance to have breast cancer will increase. Among these 71 breast cancer patients, the number of patients with breastfeeding is smaller than the patients without breastfeeding. This shows that breastfeeding might help to prevent breast cancer. The analysis of other risk factors as well. The analysis based on types of screening tests done on all patients has been summarized. The objectives for this project have been achieved through the development of analytic database management system.
